# Biotic elicitation for scopolamine production by hairy root cultures of *Datura metel*

**DOI:** 10.22099/mbrc.2017.25776.1275

**Published:** 2017-12

**Authors:** Zahra Shakeran, Mehrnaz Keyhanfar, Mustafa Ghanadian

**Affiliations:** 1 Department of Biotechnology, Faculty of Advanced Sciences and Technologies, University of Isfahan, 81746-73441, Isfahan, Iran; 2 Department of Pharmacognosy, School of Pharmacy and Pharmaceutical Sciences, Isfahan University of Medical Sciences, Isfahan, Iran; 3 Isfahan Pharmaceutical Sciences Research Center, Isfahan University of Medical Sciences, Isfahan, Iran

**Keywords:** Secondary metabolites, *Bacillus cereus*, *Staphylococcus aureus*

## Abstract

The (-)-hyoscyamine, atropine and scopolamine (hyoscine) are three valuable tropane alkaloids while scopolamine is the most important member of this group for the pharmaceutical industry due to its higher demand compared to hyoscyamine and atropine. Scopolamine is an anticholinergic reagent with several therapeutic applications. In the current study, the hairy roots culture of *Datura* *metel* was used as an advantageous method for production of scopolamine. The hairy roots are formed by *Agrobacterium rhizogenes* and have genetic stability, high growth rate and lateral branching. In this study, the effect of *Bacillus cereus* and *Staphylococcus aureus* as biotic elicitors on the production of scopolamine in *D. metel* hairy roots was investigated. The amount of scopolamine in the hairy roots was detected by HPLC analysis and compared with control samples after 0, 12 and 24 hours. Results showed that, *B. cereus* and *S. aureus* enhanced scopolamine production in the culture while the atropine content was decreased. Although in the control samples with no bacterial elicitation no scopolamine was detected, elicitation by *B. cereus* caused production of scopolamine and about 0.03 gram and 0.017 gram of it was detected in 100 gram dried *D. metel* hairy roots after 12 and 24 hours respectively. In *S. aureus* elicited hairy roots, scopolamine was not produced after 12 hours. However, about 0.025 gram of this tropane alkaloid was detected in 100 gram dried hairy roots after 24 hours. In conclusion, *S. areus* and *B. cereus* induced the scopolamine production in *D. metel* hairy roots.

## INTRODUCTION

Plants are one of the main sources of therapeutic compounds and using medicinal plants are growing rapidly across the world. One of the most important plants derived group of secondary metabolites with pharmaceutical effects is alkaloids. The (-)-hyoscyamine, atropine, and scopolamine (hyoscine) are important pharmaceutical tropane alkaloids, a class of alkaloids [1]. These tropane alkaloids are extracted mostly from Solanaceae species such as *Hyoscyamus*, *Duboisia*, *Scopolia*, *Atropa*, *Brugmansia*, and *Datura* [2]. Today, the demand for scopolamine is about 10 times higher than that for hyoscyamine and atropine [3]. While scopolamine is an anticholinergic factor and it is used to stop certain digestive problems, it is also widely exploited to treat muscle spasms and motion sickness [4]. In *D.* *metel* and *Duboisia spp*, the yield of scopolamine is greater than that of hyoscyamine [5], and the content of scopolamine in different plant species including *Duboisia myoporoides*, *Atropa belladonna* and *Hyoscyamus muticus* that produce tropane alkaloids is reported to be ranging from 0.2 to 32 mg/g DW [3]. Since the procedures such as calli or cell cultures demonstrate scant efficiency for production of scopolamine, in the current study, the hairy roots cultures were used as an advantageous method to increase the production of scopolamine in *D.* *metel* [6]. The hairy roots were formed using *A.* *rhizogenes* and have genetic stability and lateral branching as well as high growth rate. The *A.* *rhizogenes* is a Gram negative bacteria and can transform the plant’s roots to the hairy roots with Ri T-DNA plasmid [7]. The hairy root cultures are very fast growing even in a hormone free medium with relatively low cost culture requirements [8]. 

In our previous study, the formation of the hairy roots in four Solanaceae species was performed using different strains of *A. rhizogenes* [9]. Some experimental procedures such as selecting the clones which are more generative or adding biotic or abiotic elicitors to the culture medium, can improve the hairy roots’ growth and secondary metabolite production. Elicitors can induce the accumulation of various types of defense responses such as phytoalexins in the plants. The use of biotic or abiotic elicitors is one of the most common methods for improving the productivity, growth rate and secondary metabolites biosynthesis in the hairy roots [10]. 

Several reports are available in the literature that determined the effect of different elicitors on the tropane alkaloids production and gene expression. For instance, methyl jasmonate, silver nitrate and chitosan were used as abiotic elicitors and yeast extracts, fungal hyphae and some bacteria were applied as biotic elicitors for improving the tropane alkaloids production [11-12]. The biotic elicitors, *B. cereus* and *S. aureus*, and the abiotic elicitors, AgNO3 and nanosilver, selected for inducing the hairy roots, were indicated that they had some effects on atropine production and release of metabolites in these roots [13]. 

In another study, gram-positive strains such as *S. areus *were more effective in increasing the yield of tropane alkaloids in comparison to the gram gram-negative strains [12]. Biotic Elicitation mechanisms have the advantage over plant systems for the production of useful intermediates and metabolites in large quantities [14]. In this study, the effects of two biotic elicitors (*B. cereus* and *S. aureus*) were investigated on scopolamine production in the hairy roots of *D.* *metel*.

## MATERIALS AND METHODS

**Plant transformation to obtain hairy roots: **The *D. metel* seeds were germinated in ½ MS media, and the leaf explants were infected with *A. rhizogenes A4* to obtain the hairy roots [9, 15]. 

**Confirmation the presence of rolB gene in the hairy roots: **The genomic DNA of the untransformed root (as a negative control) was extracted as well as the genomic DNA of putatively transformed root using the CTAB method [16]. The DNA plasmid of *A. rhizogenes A4 *was extracted as a positive control by the Sambrook method [17]. The PCR primers (forward/reverse) with sequences of 5´-ATG GAT CCC AAA TTG CTA TTC CCC ACG A-3´ and 5´TTA GGC TTC TTT CAT TCG GTT TAC TGC AGC-3´, were used for the amplification of the *rolB* gene in Thirty-five thermal cycles (denaturation at 94°C (1 min), annealing at 58°C (1 min), and extension at 72°C (1 min) for each cycle). PCR conditions included of 2 ng of plant DNA (by a 2 μl volume), 0.4 μl of 10 mM dNTPs, 1 μl of 1 mM from each primer 0.5 μl of 5 Units/ μl Taq DNA polymerase, 2.0 μl of 10× Taq buffer, 2.0 μl of 50 mM MgCl_2_, 11.1 μl of sterile ddH_2_O which the total volume became 20 μl [18]. 

**Hairy roots cultivation and propagation: **For root cultivation, 5 cm of each transformed root was cultivated in 100 mL of hormone-free half-strength Murashige and Skoog liquid culture medium in a 200 mL Erlenmeyer flask and incubated on a rotary shaker at 120 rpm and at 27°C for 18 days. Hairy roots of *D. metel* were in the exponential phase after 18-day cultures [13]. The transformed roots were exposed to elicitors for 12 and 24 h [19].

**Selection of biotic elicitors:** Two strains of bacteria, *B. cereus* and *S. aureus* were cultured in the nutrient and tryptic soy broth liquid medium respectively and were both placed on a shaker incubator, rotated at 120 rpm with 27 °C for 1 day. These cultures of bacteria were diluted to obtain optical density of 1.0 at 600 nm. From each suspension of bacteria, 13.3 ml was added to 100 ml of 18-day-old hairy root cultures [12]. These elicited hairy roots were incubated in a rotary shaker with 120 rpm at 27 °C for 12 and 24 hours and scopolamine production were measured with triplicate repeat.

**Extraction and measurement of scopolamine**: Dried root samples were powdered and extracted with ethanol: H_2_SO_4_ (2%) for 48 hours under continuous agitation at 25°C, and then filtered, followed by adding 1mg sodium sulfate. These extracts were washed with ether and dried at room temperature. The phosphate buffer was added to every dried sample. The samples were analyzed by reversed-phase high-performance liquid chromatography according to our previously published procedure [13].

**Statistical analysis**: Each experiment was performed in triplicate. Variance was analyzed with SPSS 21 using a univariate procedure at P < 0.05.

## RESULTS

The *D. metel* leaf explants were induced by *A. rhizogenes *A4. After 15–20 days the hairy roots were supposed to be seen through these explants, especially from the regions that were inside the wound sites. The *A. rhizogenes* plays a role in integration of the T-DNA from root-inducing (RI) plasmid into the plants DNA genome. The *A. rhizogenes* *rolA*, *rolB* and *rolC* oncogenes are involved in regulation of plant growth and cell differentiation [20]. The *rolB* gene that is carried by TL-DNA, has an important role in the release of active auxin [6]. To confirm the presence of *rolB* gene in the hairy roots sample, PCR amplification was performed and this gene was emerged in 780 bp on gel electrophoresis and was compared to the positive and negative controls.

The amount of scopolamine in the control and elicited samples was analyzed using HPLC method and the content of this alkaloid was measured as the percentage of the dry weight. 

The standard samples of scopolamine were calibrated in 10, 25, 50, 100, 250, and 500 µg/ml concentrations and the obtained equitation was; y=16201x-47190. The correlation between the peak area and the concentration was analyzed with the minimum square method (R^2^ value).

**Figure 1:**
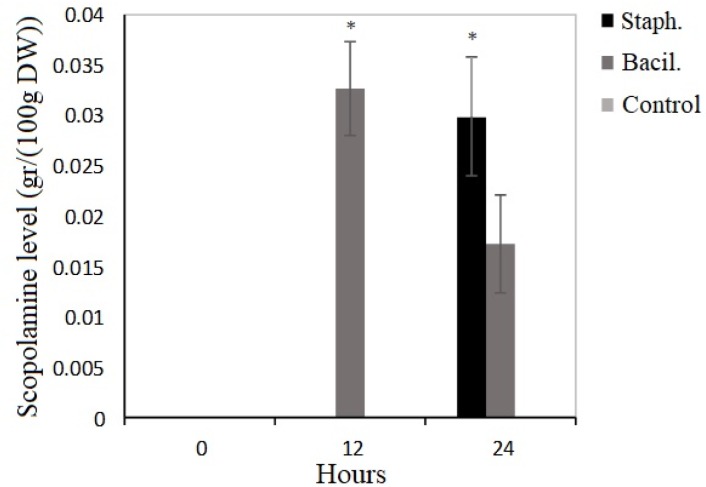
The effect of biotic (*B. cereus* and *S. aureus*) elicitors on scopolamine content in hairy roots of *D. metel*. Stars labels show significant differences in mean values for each parameter using Duncan’s test (P < 0.05).

The results indicated that no scopolamine were detected in the control samples after 0, 12 and 24 hours, while the amounts of this alkaloid were significantly increased after elicitation by *B. cereus* in 12 and 24 hours and by *S. aureus* in 24 hours to compared with controls, where P< 0.05 (Fig. 1). 

**Figure 2:**
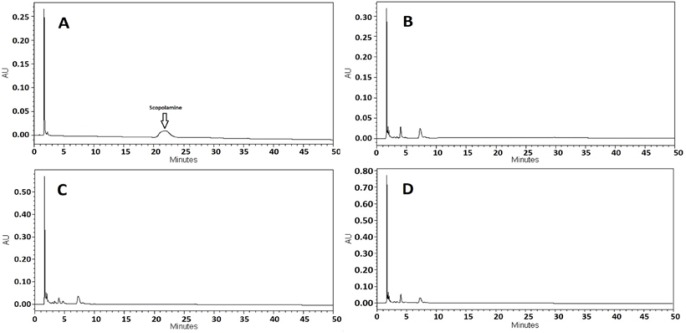
HPLC chromatograms of a) scopolamine standard (500 μg/ml); b, c and d) control hairy root samples without any elicitation after 0, 12 and 24 hours.

**Figure 3:**
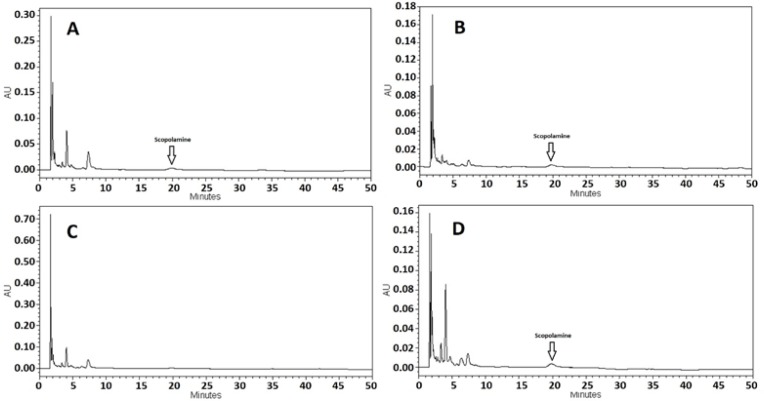
HPLC chromatograms of a and b) scopolamine from the hairy roots of *D. metel* elicited by *B. cereus* after 12 and 24 hours, respectively; c and d) scopolamine from the hairy roots of *D. metel* elicited by *S. areus* after 12 and 24 hours, respectively.

The HPLC chromatogram of the scopolamine standard (500 μg/ml) was detected in Fig. 2a. .According to our previous study, the peak of atropine, another tropane alkaloid in *D. metel*, with a retention time of about 7 min could be found in the HPLC chromatogram. This peak was detected clearly in HPLC results of control samples, however, no peak was seen in these samples, at the retention time of about 20 minutes for scopolamine (Fig. 2b, 2c and 2d). The peak of scopolamine was just found in the biotic elicited hairy root samples chromatogram. In addition, the peak of atropine in the samples that were elicited by *B. cereus* and *S. areus* decreased compared to that of the control samples in the results of HPLC analyses (Fig. 3a-d).

## DISCUSSION

The hairy roots have been investigated as a valuable source of extracting secondary metabolites from medicinal plants while this phenotype is characterized by growing in hormone free medium, genetic stability, lateral branching and low cost productivity in high scale [6]. Two important secondary metabolites in the Solanaceae family are hyoscyamine and scopolamine and these tropane alkaloids can be produced by the hairy roots systems [21]. In this study, the PCR amplification of the *rolB* gene indicated that this gene appeared in 780 bp on gel electrophoresis and was confirmed by positive and negative controls. The similar results were also obtained in the other reports [18]. 

In the current study, the hairy root cultures were inoculated and co-cultured with *B. cereus* and *S. areus* as the biotic elicitorsPlant defense mechanisms can be induced by various microorganisms, particularly the rhizobacteria such as *B. cereus* that is growing in the plants [22]. Moreover, the exogenous IAA, produced by *B. cereus*, is promoting the root growth rate at the early stage of co-culture process [23]. However, the hairy roots can inhibit the *B. cereus* growth. This effect might be due to the secondary metabolite production, induced by *B. cereus*, followed by decreased medium pH in the root-bacteria co-culture. This inhibiting effect is increased significantly by the time. The presence of *B. cereus* as the live bacteria in the culture also has an inhibitory effect on the hairy roots growth [22]. In this study, after 24 hours, little browning of the roots was initiated due to cell lysis caused by living bacteria, *B. cereus* and *S. aureus*, attack. Therefore, the weight of hairy roots was decreased[12, 22].

In the current study, no content of scopolamine could be detected in the control samples while the scopolamine was found in the hairy roots elicited by *B. cereus* and *S. areus*. Besides, our previous study showed that the atropine content was decreased in the hairy roots elicited by the same biotic elicitors comparing to the control roots [13]. Therefore, it can be concluded that, the tested biotic elicitors increased the production of the scopolamine and decreased the atropine production. 

The hyoscyamine and scopolamine are structurally related and scopolamine is 6, 7-β-epoxide of hyoscyamine formed from hyoscyamine by hyoscyamine 6-β-hydroxylase (H6H) through two steps mediated by 6-β- hydroxyhyoscyamine. Hence, when a sufficient amount of the H6H presents, the production of scopolamine is clearly detected without significant presence of 6-β- hydroxyhyoscyamine [24]. The conversion of hyoscyamine to the much more valuable scopolamine can be increased by relating the expression of key enzymatic activities. The overexpression of the *h6h* gene in the hairy roots improves scopolamine production due to more production of the H6H enzyme. In other reports, the overexpression of various genes such as *pmt*,* h6h,* *TR1 *and *TR2* associating in the biosynthetic pathway of tropane alkaloids specially in order to enhance production of scopolamine had been performed [11, 21, 25].

It has been reported that, different biotic and abiotic elicitors enhanced and transformed the production of secondary metabolites in hairy roots of some plants [26]. The effects of abiotic elicitors such as ethanol, methyl jasmonate and Ag+ on production of tropane alkaloids were surveyed in the hairy roots of *Anisodus acutangulus* and it was improved that the accumulation of tropane alkaloids is due to up-regulation of hyoscyamine-6 β -hydroxylase and increased expression of tropinone reductase I and putrescine N-methyltransferase I [11]. In addition, two gram-positive strains (*B. cereus* and *S. aureus*) and one gram-negative strain (*Pseudomonas aeruginosa*) of bacteria were used as biotic elicitors and these bacteria increased the scopolamine concentration in *Scopolia parviflora* [12]. Hence, according to the results of the current study and the similar results reported for the effect of *B. cereus* and *S*. *aureus* on the scopolamine content, it can be concluded that the bacteria caused the overexpression of *h6h* gene in the hairy roots of *D. metel* and consequently improved the conversion of atropine to scopolamine [12, 24]. However, the delay effects observing in the production of scopolamine after induction by these bacteria, especially in the samples elicited by *S. areus* could relate not only to biosynthesis of *h6h* gene in the hairy roots cells, but also associated with the production of 6-β- hydroxyhyoscyamine as an intermediate compound. 

In conclusion, the induction of the hairy root systems by biotic elicitation mechanisms, using the biotic elicitors such as *B. cereus* and *S. areus* could be an effective method for production of scopolamine as a much more valuable tropane alkaloid in *D. metel*.
